# Supplementary figures and images for: High-order functional redundancy in ageing explained via alterations in the connectome in a whole-brain model
Source: PLoS Comput Biol. 2022 Sep 2;18(9):e1010431. doi: 10.1371/journal.pcbi.1010431 (PMC9477425; doi:10.1371/journal.pcbi.1010431)

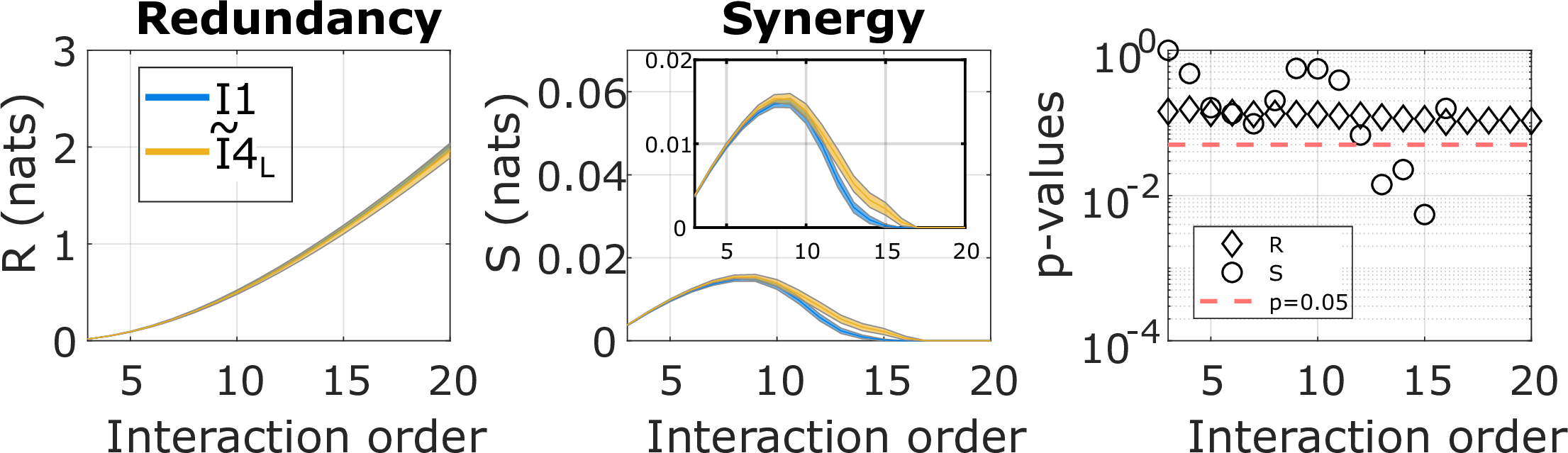

Supplement: S1 Fig — Similar to Fig 3C in the main text, but for a synthetic I4 age group using a linear model (Ĩ4L) for ageing instead of a quadratic one. Redundancy (left) and synergy (middle) are plotted as a function of interaction order and then, for each interaction order, Wilcoxon rank-sum test p-values are obtained after comparing their values between the groups Ĩ4L and I1. If the p-values survived the FDR multiple comparison correction, both the diamonds and the circles were filled in. In this case, none of the values passed the test. (TIF) [file pcbi.1010431.s001.tif]

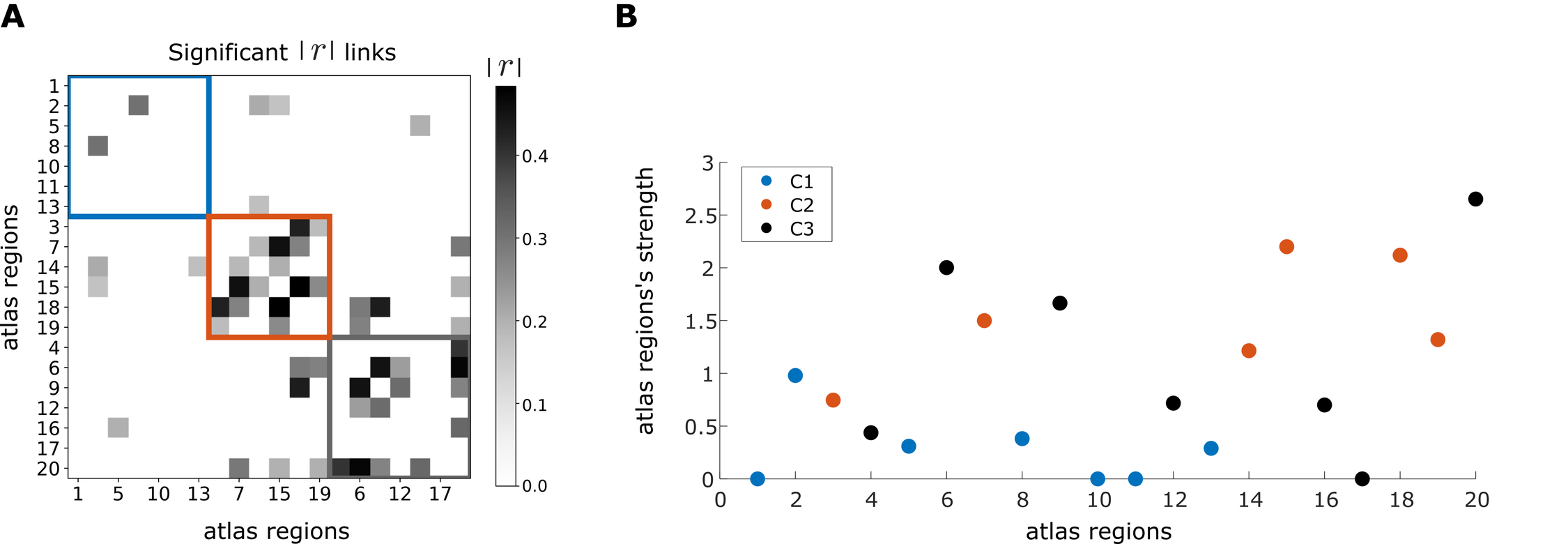

Supplement: S2 Fig — Nodes here are atlas regions. A: Rate degeneration matrix calculated using the absolute values of r, and where all links in the matrix survived to Bonferroni correction (a similar matrix was shown in the right panel of Fig 4B of the main manuscript). B: Strength values for all atlas regions (nodes) calculated on the rate degeneration matrix show in panel A. Node strength was calculated by summing over all positive values in each row or column in the matrix. A,B: Colors blue (C1), orange (C2) and black (C3) indicate different communities. (TIF) [file pcbi.1010431.s002.tif]

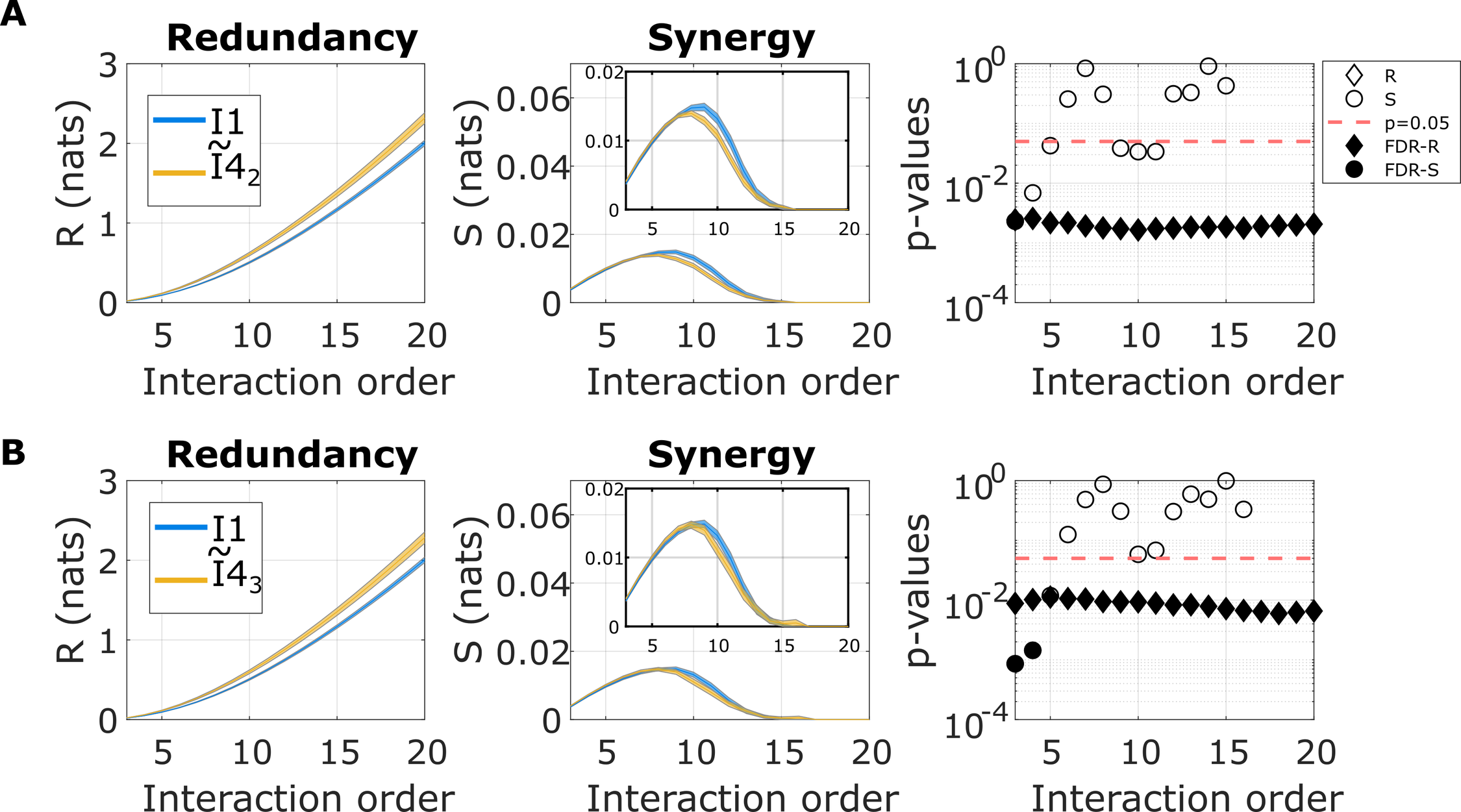

Supplement: S3 Fig — Similar to Fig 3C, here we compare redundancy and synergy by synthetic aging of the I2 and I3 groups using the quadratic aging model, giving respectively the aged groups Ĩ42 (panel A) and Ĩ43 (panel B). When comparing redundancy and synergy of these two groups with group I1, both situations provided significant redundancy differences after FDR multiple comparisons (filled diamonds) across all interaction orders. (TIF) [file pcbi.1010431.s003.tif]
