# Supplementary material for: High-order functional redundancy in ageing explained via alterations in the connectome in a whole-brain model
Source: PLoS Comput Biol. 2022 Sep 2;18(9):e1010431. doi: 10.1371/journal.pcbi.1010431 (PMC9477425; doi:10.1371/journal.pcbi.1010431)
Supplement: Attachment — Submitted filename: rrf.pdf [file pcbi.1010431.s004.pdf]

Response to Reviewers' Comments
Manuscript ID: PCOMPBIOL-D-21-01766
"High-order functional interactions in ageing explained via alterations in the connectome in a whole-brain model"

Marilyn Gatica, Fernando E. Rosas, Pedro A.M. Mediano,
Ibai Diez, Stephan P. Swinnen, Patricio Orio, Rodrigo Cofré, Jesus M. Cortes

April 12, 2022

We would like to thank the editor and the two reviewers for their helpful comments and suggestions, which have allowed us to greatly improve our manuscript. We have prepared a revised version of the manuscript, which addresses the reviewer's concerns and incorporates their suggestions. The major additions are:

- A new figure 4 in the main text, showing the relevant communities related to the brain degeneration of weights calculated via Spearman's non-parametric rank-correlation — instead of the previously used Pearson's correlation.
- A new supplementary figure 1, showing that the linear model of ageing did not give rise to significant differences in redundancy between simulated brain signals of different age groups.
- A new supplementary figure 2, showing node strength values for the three communities affected by age.
- A new supplementary figure 3, showing the results of redundancy and synergy along different interaction orders when comparing I1 with I4 when aging with a quadratic model the groups I2 and I3 instead of I1.
- A new sub-section focused on statistical analyses has been incorporated in Methods, detailing the statistics used and the employed method to correct for multiple comparisons.

In the sequel, we provide detailed responses to each of the concerns raised by the reviewers. **Text in Bold** is used to highlight the reviewer's comments, while *Text in Italics* is used for our replies. Edits in the revised version of our manuscript have been highlighted in color red.

Reply to Reviewer 1

The paper "High-order functional interactions in ageing explained via alterations in the connectome in a whole-brain model" focuses on the study of the biological (or mechanistic) origin of high-order dependencies measured using informational synergy and redundancy (more precisely, the O-information framework recently proposed by Rosas et al.). In the paper a whole-brain model is built using state-of-the-art techniques using structural and functional connectivity (fMRI) data from a cohort of subjects of age varying from 10 to 80 years. In particular, the authors fit the computational model to the standard functional connectivity matrix (FC) as it is conventionally done, and then measure the high-order properties of the resulting synthetic time-series, uncovering a dependence of synergy and redundancy similar to that observed in data in a previous publication by some of the authors (namely a growth of redundancy with the order interactions and a peak for synergy at an intermediate order). To show that the origin of these properties lies in the brain's structural degeneration, they aged artificially the young structural connectomes (using a quadratic model fit) and simulated again the corresponding functional connectivity, showing again similar results to the ones previously obtained for real data. Finally, motivated by the effects of structural degeneration, the authors use a standard community detection algorithm to extract communities on a new adjacency encoding the correlation between age and degeneration for each link, finding two main communities. The paper is original and reports its central concept quite nicely. The language is clear and the explanations can be easily followed overall. I do however have several conceptual and technical concerns that I would like to see addressed before I can consider this contribution appropriate for publication in plos computational biology.

We thank the reviewer for the very positive feedback, and for all the constructive comments and suggestions.

My first comment is conceptual: why should we fit the FC matrix alone instead of directly the set of higher-order redundancy/synergy values? Is this to state that pairwise correlations are sufficient to describe all the higher-order dependencies? But if so then, why bother with them if fixing the FC statistics is already sufficient to reconstruct the high-order ones?

We thank the reviewer for raising this very interesting observation. The aim of this article is to show that differences in high-order functional interactions between age groups are directly related with variations in the connectome. The motivation behind fitting the FC matrix alone was twofold: i) it is widely used as a general-purpose fit metric in many whole-brain modelling papers; and ii) it is a more 'basic' quantity than e.g. the O-information. Therefore, the fact that the DMF model fitted only with pairwise FC is able to capture the high-order differences between groups is a positive and non-trivial result. If, conversely, we had fit the O-information values directly, it would be unsurprising to see the empirical results reproduced, as they would have been forced onto the model explicitly. Furthermore, it is important to emphasize that the observed changes in high-order interactions are surprising and not trivial, as we show are related to a quite specific type of change in the connectome. In particular, our results suggest the need of including non-linear dependencies in the connectome aging process to reproduce these high-order effects in the DMF modelling. The discussion section of our revised manuscript includes comments and clarifications related to these important issues.

The paper states that the DMF (Figure 2, and the "aged" young connectomes in Figure 3) reproduce the observed patterns from ref 24. However, a fairer assessment would be that the synthetic time-series show a qualitatively similar behaviour to the original data, e.g. synergy has a peak for intermediate order, while redundancy keeps growing with the order. Quantitatively, however there seems to be a considerable discrepancy, e.g. the synergy peak in data is around order 12, while in simulations it's around 7; the values of redundancy and synergy too seem overall much smaller than those found in the data (e.g. synergy peaks at $\tilde{0}.05$ in simulations, but above 0.1 in data) and so on. I wonder whether this is a question of different scales across the two paper, but I think it would be very important to plot the R/S values from data in Figure 2 at least, for comparison to those obtained from the synthetic time series.

We thank the reviewer for pointing out this relevant point. Indeed, the reviewer is correct in stating that the precise values of R and S are quite different from the values reported in Ref. 24. However, we want to emphasize that main objective of our study was to show that the modeling explains the statistically significant differences between age groups at all interaction orders and not reproducing precise values. This now has been incorporated into the manuscript.

Going deeper into this concern, and following the reviewer's suggestion, we have evaluated the synergy-to-

redundancy (S/R) ratio in the two cases, empirical data (A, results in Ref. 24) and DMF (B, modeling approach), and get:

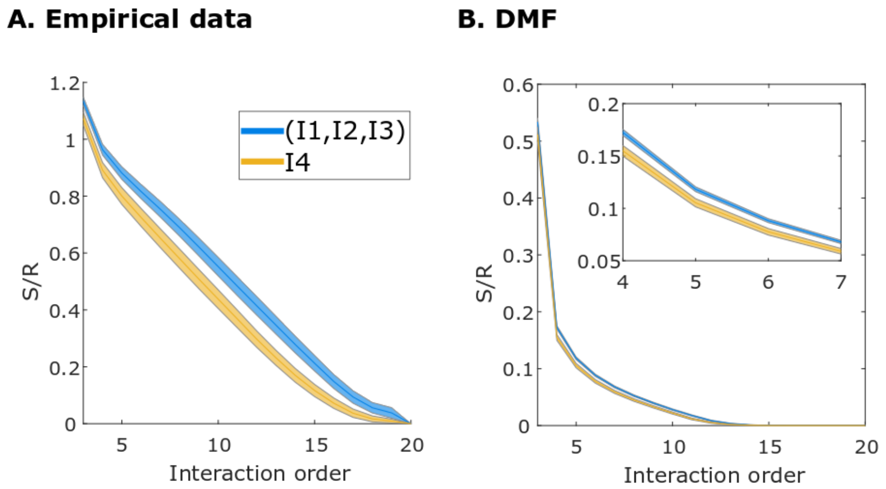

Although the two plots follow similar trends along interaction orders for both groups I4 and (I1,I2,I3), the empirical S/R ration is approximately twice that of the DMF. Furthermore, the transition from interaction order 3 to 4 is more abrupt in DMF compared to the empirical one. Overall, and returning to the previous point of the reviewer, our modeling approach including pairwise FC fitting does not explain all the quantitative details of the interactions in R and S at higher levels.

I did not find the details about the linear model to fit the structural degeneration mentioned in the main text and of the comparison of its O-information to the ones obtained by the DMF and the quadratic model.

We would like to apologize for not having incorporated these results in the previous version. We now have included these results as a new supplementary figure S1.

Why after showing that a non-linear model is needed to link age and structural degeneration, a linear correlation measure was used to build the matrix used for community detection in figure 4? Wouldn't a rank-correlation be more appropriate? How would that change the reported results?

We would like to thank the reviewer for making us clarify this important issue. We use linear Pearson correlations because when we look at the dependency of weight versus age across different participants (see, for example, the center plot in Figure 4A), the linear dependency captures well that ageing-related decay. (and this typically occurred for all weights). As the reviewer correctly mention, there exist non-linear dependencies as shown in figure 3A (referred to as connectome degeneration), which explain the dependence of the weights in the I4 group (older participants) as a function of the weights in the I1 group (younger group). It is precisely here where the linear fit does not provide significant differences between age-group (this is shown in the new supplementary figure S1) but the quadratic fit does. However, and following the reviewer's suggestion, we have repeated the same analyses but using the Spearman's non-parametric rank-correlation, known to perform better in the presence of outliers. The new figure 4 has been completely revamped, but now using the Spearman's correlation. As the reviewer can see, comparing the new figure 4 with the old one, although small differences occurred for some links in the matrix, after multiple comparisons, the significant links that were preserved remained almost unchanged.

Community detection 1: why was Louvain used? More precisely, what was the matrix given as input to the Louvain algorithm? the actual signed matrix or its positivised version? This is important, because, to my understanding, in its standard formulation (like in BCT), modularity (and the Louvain algorithm) accepts weighted graphs but it tries to maximise the sum of edge weights within a module and minimize that of links in between modules. The paper says that most edges (Fig. 4) are negative, thus the communities found should be those that have the smallest negative values inside. However, the plot reports the absolute value of r, and the block-reordered version (Fig. 4b) shows credible blocks. can you please provide a bit more detail?

We would like to thank the reviewer for raising all these different points. Why do we use Louvain's modularity? Although the final adjacency matrix (constructed by the correlation between the individual weights and the age of the participants) is not large and has a dimension of 20×20, community detection was applied to reduce the dimensionality while studying the emergent properties in the aggregate or module level (thus eliminating

the need. to study the relation between weight with age at the level of a single link). The choice of Louvain community detection was used because it has been widely used and has demonstrated multiple computational advantages in network community detection compared to other methods. Namely, Louvain's method is very easy to implement following simple heuristic rules and provides a computationally fast approximate solution to the NP-hard problem of optimizing modularity.

However, despite computational advantages of the Louvain's method, it is sometimes problematic resulting in a set of communities that are highly unstable, ie., difficult to obtain by other methods, and dependent on the parameter values used to calculate the modularity metric. In particular, the final solution may depend on the chosen resolution parameter γ (in our work set to one, thus weighing equally the actual number of edges in a community and the expected number of edges in the same community). In our results, this is not the case, as tuning γ from 0.9 to 1.2 (in steps of 0.1) the solution of final communities preserved quite well with a cluster integrity ≥ 0.8 (as measured by the Spearman rank-correlation between the two solutions, the one we show in our results for $\gamma = 1$ and and the corresponding one for the different values of γ). In the figure below we plot the cluster integrity for different values of γ.

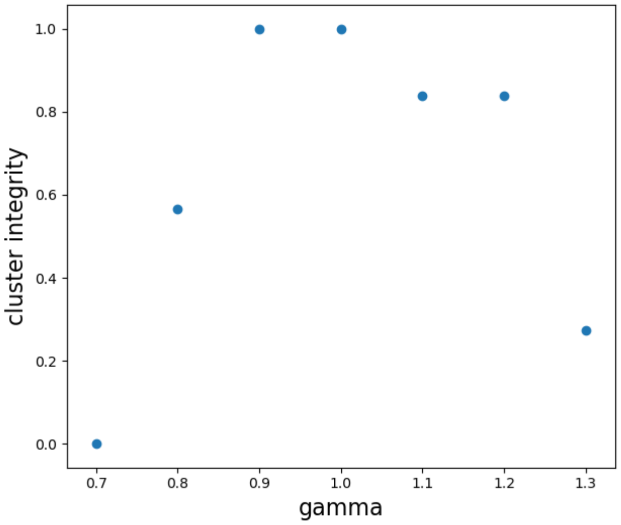

Continuing with this concern, the reviewer is right that for modularity maximization, one needs to assess the intra- and inter-module strength, and in the case of matrices with values between -1 and 1, the calculation of the strength is problematic, since it have a bias towards solutions of lower value due to the compensation between different sign values. We have applied Louvain's modularity detection to the absolute value matrix –we acknowledge that this was not clearly explained in the previous version of the manuscript– , and as the reviewer acknowledges, this is why the graph on the right in figure 4B shows coherent modules. All these points have been clarified now in the new manuscript.

Community detection #2: why modularity? Modularity always gives a result even when it's not statistically justified or below the resolution limit. Have you tried reproducing the results using different community detection algorithms (e.g. stochastic block models), which although different in nature, have also the possibility of returning more general architectures than block structures and -when appropriate–no blocks at all?

The reply to this point is somehow overlapping with our previous answer. More importantly, the fact that our final communities are preserved for different values of the resolution parameter γ (see figure above), indicates that our communities are quite stable and detectable under different initial conditions and optimization trajectories. These results on the stability of the communities found by the Louvain's method have now been incorporated into the manuscript.

Node properties: node strength with reference to the matrix defined in Fig.4 is used multiple times in the text. How are these strengths computed (in light of the fact that all edge weights are negative)?

We apologize for not being clear enough in the previous version of the manuscript. The strength was calculated in the matrix of absolute values of the significant links obtained by correlation (in the new version of the manuscript

we have used Spearman's correlation as suggested by the reviewer; while in the previous version of the manuscript we used Pearson's correlation). The strength, therefore, obtained by summing only over positive values, is a good metric in relation to the centrality role that a given node is playing in a network. As the reviewer can see in the following figure, the strength of the node is ≥ 0*, since some nodes do not have any significant link to any other node. Furthermore, comparing Spearman's correlation with Pearson's, we have now obtained two significant links within cluster 1 (C1), which did not exist when the links were Pearson-calculated. The other two clusters C2 and C3 remained similar to those obtained using Pearson correlation. This has now been clarified in the manuscript and we have included a new supplementary figure S3 calculating all node strength values in the matrix of absolute values of Spearman's correlation.*

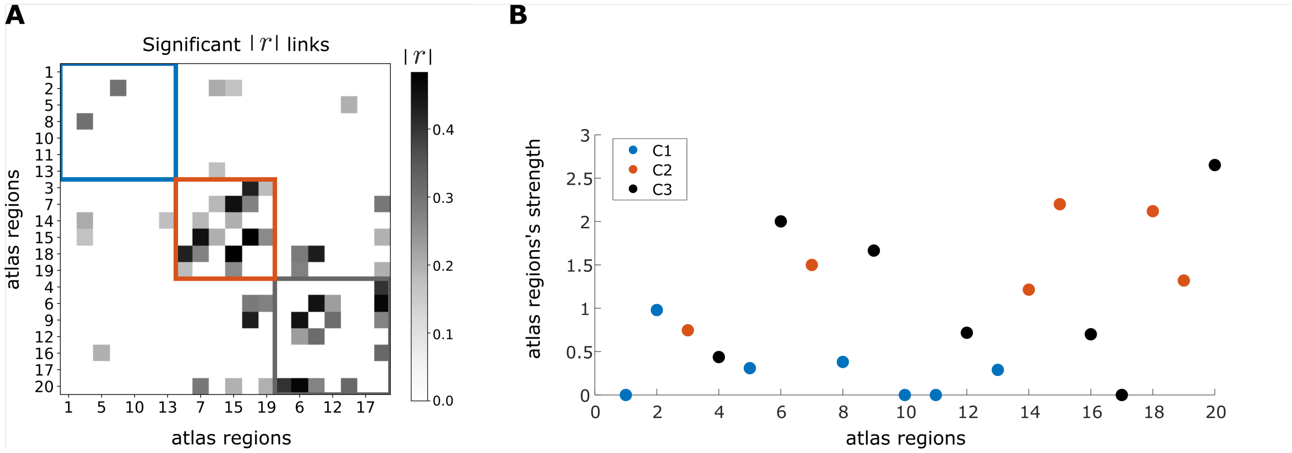

Statistical comparison: multiple times in the text multiple comparisons and corresponding corrections are mentioned but no statistics are reported for the tests, how they were performed, etc. Can you please provide additional details?

We would like to thank the reviewer for this suggestion. The statistic used was the non-parametric Wilcoxon rank-sum test, while the correction for multiple comparisons was applied using False discovery rate (FDR) following a standard Benjamini-Hochberg procedure. We have now added a new Statistical analysis section in Methods with this information. Additionally, we now provide the rank-sum statistic (RS) values along with the p-value in the results, as suggested by the reviewer.

Reviewer 2

I am attaching my report on the manuscript entitled: High-order functional interactions in age ageist explained via alterations in the Connectome in a whole-brain model, by Gatica et. Al. In this work, the authors address ageing under a high-order functional perspective. Inspired by a recent empirical result in high order interactions on functional brain networks (ref. 24, where high order metrics were empirically proven to be a biomarker of ageing), the authors developed a realistic model - whole brain dynamic mean-field model as well as Haemodynamic model - which includes both functional and structural connectomes. By applying the model to the youngest age group (I1) and comparing it to the oldest group (I4), the model reproduced the findings in ref. 24 on high order interactions, i.e., similar age variations in high order interactions of redundancy and synergy. The paper is well written, and the results are sound. Yet, the authors made their codes available for reproducibility purposes, and I would like to commend them for that. Ageing is a relevant topic per si, and coming with a model that reproduces high order aspects of ageing may impact the next steps of high order neuroscience. Based on the report above, I believe that the manuscript fulfil the relevance and quality criteria for publication in PLOS computational biology. However, before publication, I think that the authors should address the following (minor) points:

We thank the reviewer for the very positive comments and feedback.

Since the manuscript is based on the findings of ref. 24, for self-consistently purposes, I would summarize a bit more the results of ref. 24 in this manuscript. I knew ref. 24 in advance, but a reader not familiar with ref. 24 may not follow this manuscript.

Thank you, this is an excellent suggestion. In the new version of the manuscript we briefly summarize the results of ref. 24 in the third paragraph of the introduction.

The authors used the model to predict High order metrics of an "aged version" of group I1 and compared it with group I4. Would those findings be doable (or applicable) for intermediate groups (I2 and I3)? Since the authors mentioned future work in other forms of brain degeneration, some disorders (e.g. Multiple sclerosis) may start at ages in groups I3 (or even I2).

We would like to thank the reviewer for this very constructive suggestion. The following figure, now included in the manuscript as new supplementary figure S3, shows the results of the suggested analysis. We compare the results of group I1 with synthetic aging groups I2 (upper row) and I3 (bottom row), represented respectively by ($\tilde{I4}_2$) and ($\tilde{I4}_3$). In both cases, significant redundancy differences are found after multiple comparison corrections. With thanks to the reviewer, we present these results as a new supplemental figure S3.

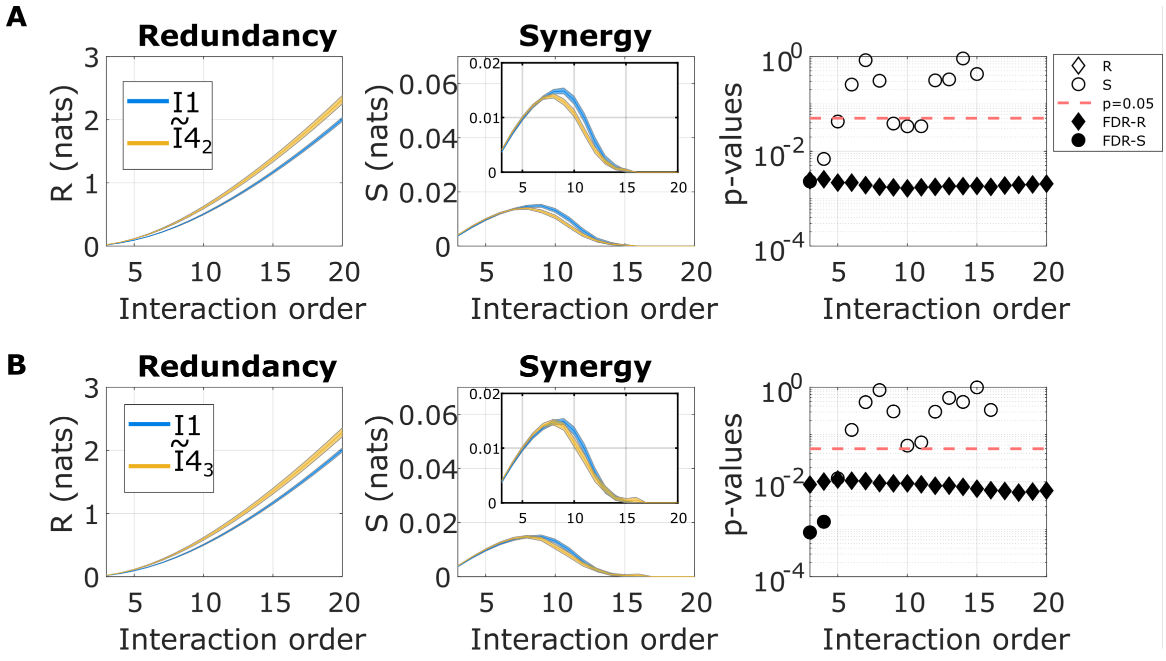

The authors assessed the relationship between each individual SC link and age. They computed the Pearson correlation between SC matrix and age to do so. However, It's known (and also mentioned in this manuscript) that multiple properties related to ageing are nonlinear and often quadratic. The authors used a quadratic fitting in Fig. 3A to model Connectome degeneration between group I1 and I4. Therefore, as a posthoc analysis, I would suggest the use of connectivity metrics that can capture eventual nonlinear relations between the SC links and age (e.g. mutual information), which could be more appropriate than Pearson correlation (which captures only linear relationships between variables).

We fully agree with the reviewer concern. At different levels, from morphology and circuitry to cognitive and behavioral performance, different metrics along lifespan follow nonlinear trends. This is shown in figure 3A (referred to as connectome degeneration), which explains the dependence of the weights in the I4 group (older participants) as a function of the weights in the I1 group (younger group). However, we would like to mention that Pearson's correlation was used because when you look at the dependency of weight versus age across different participants (see, for example, the center plot in Figure 4A), the linear dependency captures well that aging-decay (and this typically occurred for all weights). Following the recommendation of another reviewer, who raised a similar point, we have repeated the same analyses using Spearman's non-parametric rank-correlation. New figure 4 has been completely revamped, but now using the Spearman's correlation. As the reviewer can see, comparing new figure 4 with the old one, although small differences occurred for some links in the matrix, after multiple comparisons, the significant links that were preserved remained almost unchanged. Regarding the use of non-linear measures of dependency, as suggested by the reviewer, the following figure presents the corresponding results for Mutual Information, which is in agreement with the results of new figure 4 obtained using Spearman's correlation.

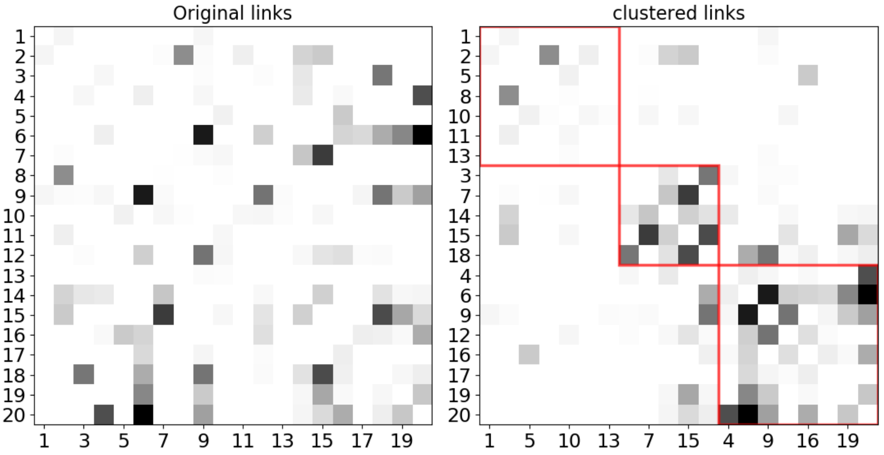

The reason why the results of Mutual Information are similar to those of Spearman could be because the high-order metrics were calculated under the Gaussian copula approximation. In the case of making non-linear estimates of these metrics, Mutual Information and Spearman may differ, but this is beyond current scope. Future studies should explore all these possibilities. These comments have been added to the discussion of the manuscript.

The fact that the model is pairwise but still captures high order interactions is quite intriguing. It would be interesting if the authors could develop or discuss a bit more on this result. Where do the high order interdependencies come from? Are they possibly coming due to the inclusion of two connectivity modalities (SC and FC)? Or from the non-linearities of the model?

We thank the reviewer for raising this very interesting observation. We believe the key factor that makes these interdependencies appear are the non-linear dynamics of the model, that generate high-order interdependencies over time as the model navigates a complex set of attractors. Furthermore, note that not any change in SC seem to suffice to recover the high-order changes between young and old participants observed in empirical data: a quite specific, non-linear SC ageing model seem to be required, as a linear aging procedure results in non-significant effects. We have added clarifications related to this important issue in the revised version of the discussion. Additionally, we have included a new supplementary figure S1 that illustrates how pairwise fitted interactions and linear aging do not reproduce the desired results.

There is another interesting result in ref. 24 that the authors did not report a comparison with the model developed here. The authors of ref. 24 reported the presence of a "redundancy core" in rs-fMRI and that this core changes as you age. In fact, the authors noted that the redundancy core of groups I1, I2 and I3 are different (and bigger) than group I4. Are these results also found in the model proposed here?

We would like to thank the reviewer again for the constructive suggestion and the interest in our work. Following this suggestion, we have performed this analysis to check whether our new modeling approach can reproduce the redundant core results reported in Ref. 24. This is illustrated in the following figure, in the left panel for the group (I1,I2,I3) and in the right panel for I4:

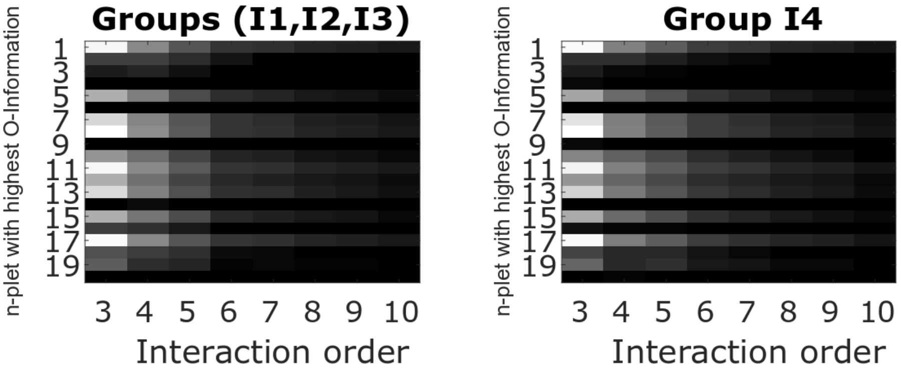

As the reviewer can see, and comparing with the results in Ref. 24, our pairwise-fitted FC modeling approach is not able to distinguish a redundant core for the I4 group, as it predicts similar redundancy sets for the two scenarios (left and right panels of the figure). Multiple reasons could explain this discrepancy between the model and the real data (Ref. 24). The main one, already recognized by both reviewers, is that we built the model by fitting FC pairwise interactions. As shown in our previous answers above, we also show that although the pairwise fitted FC model reproduces differences in the redundancy of I4 compared to that of (I1,I2,I3), the exact values of R and S cannot be either reproduced.

Furthermore, as we have also mentioned before, the pairwise-fitted FC model alone was not sufficient to reproduce these group high-order differences, since the pairwise fit with linear aging dynamics did not reproduce the results in Figure 3, and it was necessary to introduce quadratic dependencies for aging. Finally, another possible reason could be that we have an average connectome per age group, which provides a global parameter G per group that we use in all our simulations. It is possible that fitting a different model for each participant could introduce more heterogeneity, which could perhaps help improve the precise match between the actual data results and our modeling approach. We also fully agree with the reviewer, and would like to thank the reviewer for this suggestion, that building models by fitting the structure of higher-order functional interactions beyond pairs is of great interest to explore in future work, likely providing a better fitting to the data and perhaps also allow the model to make novel predictions, thus opening up new and exciting possibilities. All these comments have been added in the discussion of the manuscript.
